# Transverse Susceptibility as a Biosensor for Detection of Au-Fe_3_O_4_ Nanoparticle-Embedded Human Embryonic Kidney Cells

**DOI:** 10.3390/s130708490

**Published:** 2013-07-03

**Authors:** Natalie Frey Huls, Manh-Huong Phan, Arun Kumar, Subhra Mohapatra, Shyam Mohapatra, Pritish Mukherjee, Hariharan Srikanth

**Affiliations:** 1 Department of Physics, University of South Florida, Tampa, FL 33620, USA; E-Mails: natalie.frey.huls@gmail.com (N.F.H.); pritish@usf.edu (P.M.); 2 Department of Internal Medicine, University of South Florida, Tampa, FL 33620, USA; E-Mails: smohapa2@health.usf.edu (S.M.); smohapat@health.usf.edu (S.M.); 3 Department of Medical Laboratory Sciences, University of Delaware, Newark, DE 19716, USA; E-Mail: arunk@udel.edu (A.K.)

**Keywords:** magnetic nanoparticles, cells, magnetic biosensors

## Abstract

We demonstrate the possibility of using a radio-frequency transverse susceptibility (TS) technique based on a sensitive self-resonant tunnel-diode oscillator as a biosensor for detection of cancer cells that have taken up magnetic nanoparticles. This technique can detect changes in frequency on the order of 10 Hz in 10 MHz. Therefore, a small sample of cells that have taken up nanoparticles when placed inside the sample space of the TS probe can yield a signal characteristic of the magnetic nanoparticles. As a proof of the concept, Fe_3_O_4_ nanoparticles coated with Au (mean size ∼60 nm) were synthesized using a micellar method and these nanoparticles were introduced to the medium at different concentrations of 0.05, 0.1, 0.5, and 1 mg/mL buffer, where they were taken up by human embryonic kidney (HEK) cells via phagocytosis. While the highest concentration of Au-Fe_3_O_4_ nanoparticles (1 mg/mL) was found to give the strongest TS signal, it is notable that the TS signal of the nanoparticles could still be detected at concentrations as low as 0.1 mg/mL.

## 1. Introduction

Superparamagnetic nanoparticles (5–150 nm) have found applications in biomedicine due to their biocompatibility and to the fact that their dimensions are smaller than or comparable to those of cells (10–100 μm), viruses (20–450 nm), and proteins (5–50 nm) [1–3]. The ability of these nanoparticles to be manipulated by an external magnetic field makes them especially attractive for localized treatment options such as targeted drug delivery and hyperthermia, as well as diagnostics like enhancing contrast in existing magnetic resonance imaging (MRI) techniques and sensors based on the detection of a magnetic signal [4,5].

Iron oxide superparamagnetic nanoparticles (Fe_3_O_4_) have been examined for various biomedical applications due to their biocompatibility, magnetic properties (high saturation magnetization), and their ability to be functionalized [6–9]. However, if the surface is left untreated, agglomeration can occur, and the natural hydrophobicity of the surface causes the particles to be taken up by the body's systems, mainly the Kupffer cells in the liver [7]. Usually, Fe_3_O_4_ particles must first be coated with an amphiphilic polymeric surfactant such as polyethylene glycol (PEG) to keep them from agglomerating, and to minimize unwanted protein adsorption. The subsequent coating can then be functionalized by attaching carboxyl groups or specific molecules. It has been shown that coating the Fe_3_O_4_ particles with a noble metal, such as gold (Au), can serve a similar purpose, but in this case ‘linker’ molecules with functionalities at both ends and an affinity for Au can be used to aid in functionalization. A well-known example is thiol adsorption, in which alkanedithiols are covalently attached to the Au surface [8]. This opens up a great realm of possibilities as thiols can be bonded with proteins, peptides, carbohydrates, lipids, and DNA [8,9]. It is widely thought that Fe_3_O_4_ nanoparticles can achieve greater functionality through coating with Au and exploiting Au-thiol chemistry.

To sense cells that have taken up magnetic nanoparticles, different types of magnetic sensors based on giant magneto-resistance (GMR), spin valves, and the Hall effect have been proposed [10–15]. Among these sensors, those based on GMR technology have been widely applied to many practical problems including biosensing systems [13,15]. GMR sensors detect the presence of magnetic nanoparticles as magnetic labels via a change in sensor resistance at a fixed sensing current. The major drawback of the GMR sensors is that high magnetic fields (up to 1 T) are needed to change the material's resistance [15] and the equipment for producing GMR sensors is quite complicated which makes them expensive. In this context, the discovery of a so-called giant magneto-impedance (GMI) effect, which refers to a large change in the AC impedance of a magnetic conductor subject to a DC magnetic field, in a number of soft ferromagnetic materials, together with a very high sensitivity at low fields, lack of hysteresis and high temperature stability holds greater promise in field sensing [16–24]. Since GMI can be achieved at a small DC magnetic field (around 10^−5^ T), the field sensitivity of a GMI sensor is about 500 times higher than that of a GMR sensor [24]. This makes a GMI sensor very promising for biosensing applications. However, the development of GMI-based biosensors is in its infancy and further efforts in optimizing these sensors' performance are needed [24].

In this paper, we have demonstrated the new possibility of using a tunnel diode oscillator (TDO)-based radio-frequency (RF) transverse susceptibility technique as a biosensor for detection of Au-Fe_3_O_4_ nanoparticles taken up by human embryonic kidney (HEK) cells. Since this resonant method can detect small changes in the magnetic signal of even small amounts of nanoparticles taken up by cells, it is a very promising tool for biosensing applications.

## 2. Experimental Section

**Figure 1.**
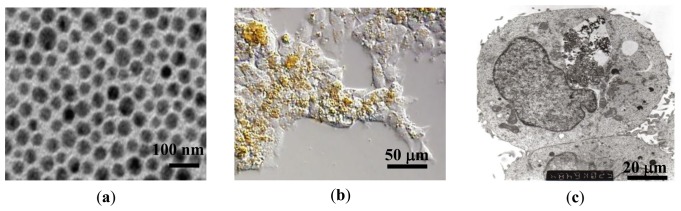
(**a**) TEM image of Au-Fe_3_O_4_ nanoparticles; (**b**) Optical and (**c**) TEM images of the nanoparticles (circled) inside HEK cells.

Au-coated Fe_3_O_4_ nanoparticles were synthesized using a micellar method by following the procedure outlined by Mandal *et al.* [25]. First, a stock solution was made by dissolving ferric ammonium sulfate (0.128 M with respect to the Fe(III) ion) and ferrous ammonium sulfate (0.064 M with respect to the Fe(II) ion) in 100 mL 0.40 M aqueous sulfuric acid. A separate solution of 1.0 M NaOH was added to 0.01 M poly(oxyethylene)isooctyl phenylether (TX-100) to make a concentration of 0.01 M TX-100. This solution was heated to 70–80 °C, and 25 mL of the iron stock solution was added drop by drop while stirring. Heating and stirring continued for 30 min while Fe_3_O_4_ nanoparticles were formed. The particles were centrifuged to separate them from the solution and washed. The resulting Fe_3_O_4_ particles were then coated with Au. For this step 0.5 g of glucose was added to a solution of 1:1 molar ratio Fe_3_O_4_ to HAuCl_4_. The solution was sonicated for 15 min and then heated at 80 °C in a water bath for 1 hour. Mandal *et al.* reported that the glucose helps promote Au-Fe_3_O_4_ adhesion and maintain uniform thickness of the Au layer [25]. X-ray diffraction measurements were performed on the bare Fe_3_O_4_ and Au-Fe_3_O_4_ particles, confirming the inverse spinel ferrite structure and fcc phase of Au. The absence of the diffraction peaks for Fe_3_O_4_ phase in Au-Fe_3_O_4_ particles was also observed, providing strong evidence for the coverage of the iron oxide core by the gold shell. The Au-Fe_3_O_4_ particle sizes of 60 nm ± 10 nm were measured using TEM (Figure 1(a)). This size of particle is desirable because the cells to be used in this experiment preferentially take up particles of 60–70 nm. The Au-Fe_3_O_4_ particles were then used for cell transfection, and subsequently tested for detection using transverse susceptibility.

Human embryonic kidney (HEK293) cells were obtained from the American Type Culture Collection (ATCC). Cells were cultured on a plastic substrate at 37 °C in minimum essential medium containing 10% fetal bovine serum and 100 units/mL each of penicillin and streptomycin in an atmosphere of 5% CO_2_/95% air. Au-Fe_3_O_4_ nanoparticles were introduced to the medium at concentrations of 0.05 mg/mL, 0.1 mg/mL, 0.5 mg/mL, and 1 mg/mL, where they were phagocytosed by the cells. Cells were then detached from the substrate by removing excess medium, rinsing the cell layer with 0.25% (w/v) trypsin-0.53 mM EDTA solution and incubating them 3–5 min with trypsin-EDTA solution. A complete growth medium was then added to the cells for incubation. Figures 1(b,c) show optical and TEM images of cells after uptake of nanoparticles. The circle indicates the region where the nanoparticles are located, and the particles appear as dark, filament-like structures. The nanoparticles can be recovered from the cells through homogenization. The particles are being phagocytosed or endocytosed at maximum concentration (1 mg/mL) of approximately 70%.

## 3. Results and Discussion

### 3.1. DC Magnetic Measurements

**Figure 2.**
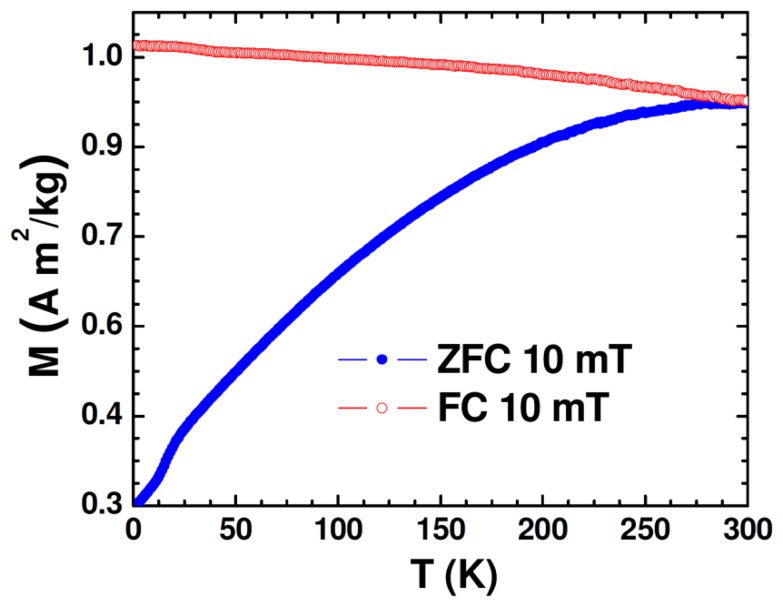
Zero-field-cooled (ZFC) and field-cooled (FC) magnetization curves (M-T) for Au-Fe_3_O_4_ nanoparticles.

The DC magnetic properties of the synthesized nanoparticle samples were studied using the physical property measurement system (PPMS) from Quantum Design (San Diego, CA, USA) equipped with a variable field (−7 to +7 T) superconducting magnet, which has a variable temperature (2−400 K). Figure 2 shows the temperature dependence of zero-field-cooled (ZFC) and field-cooled (FC) magnetization (M-T) of the Au-Fe_3_O_4_ nanoparticles.

While the FC magnetization gradually increases with lowering temperature, the broadened ZFC M-T curve is consistent with the polydisperse nature of the magnetite nanoparticles with the associated distribution in particle size and individual anisotropy axes [26]. Because the Au-Fe_3_O_4_ particles were packed into a gelatin capsule for measurement, the broadening is also likely due to dipolar interactions among particles. That the maximum of the ZFC curve occurs right around room temperature is an early indication that particles reach their maximum susceptibility at this temperature, making them ideal for sensing applications. The ZFC curve can also indicate onset of the blocking transition (T_B_ ∼ 290 K), at which the Au-Fe_3_O_4_ system begins to enter the superparamagnetic state from the ferromagnetic (blocked) state with increasing temperature. This transition temperature can further be approximated from the temperature at which the coercivity of the magnetization *versus* temperature curve goes to zero. The low-temperature feature noticed in M-T curves at ∼17 K could be associated with the presence of the Verwey transition. It was reported previously for Fe_3_O_4_ nanoparticles that as particle size was decreased from 150 nm to 5 nm, the Verwey temperature shifted down to 20 K for 50 nm particles and was no longer observable for smaller particles [27]. Given that the average diameter of Fe_3_O_4_ nanoparticles in the present study is around 60 nm, the observation of the Verwey transition at ∼17 K is very reasonable.

**Figure 3.**
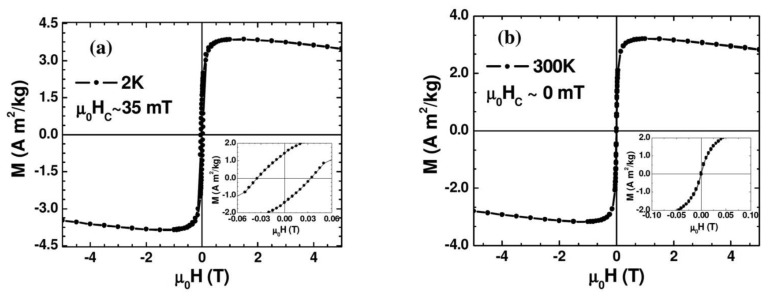
Magnetization *versus* magnetic field (M-H) curves taken at 2 K (**a**) and 300 K (**b**) for Au-Fe_3_O_4_ nanoparticles.

Figure 3 shows the magnetic field dependence of magnetization (M-H) measured at 2 K and 300 K. As one can see clearly from the figure and its inset, at 2 K the Au-Fe_3_O_4_ system shows ferromagnetic behavior with a distinct coercivity (μ_0_H_C_ ∼ 35 mT), while the coercive field appears almost zero (μ_0_H_C_ ∼ 0 mT) at 300 K where the Au-Fe_3_O_4_ system is entering the superparamagnetic state. The slight decrease of the magnetization with magnetic field for the M-H curve taken at 300 K arises mainly from the diamagnetism of Au whose contribution becomes significant at high magnetic fields (μ_0_H > 1 T). We note that the μ_0_H_C_ value of the Au-Fe_3_O_4_ nanoparticles (μ_0_H_C_ ∼ 35 mT) taken at 2 K (in the ferromagnetic state) is about twice smaller than that of bare Fe_3_O_4_ nanoparticles (μ_0_H_C_ ∼ 70 mT). This clearly shows that coating the Fe_3_O_4_ particles with Au achieves the important goal of separating the particles and reducing the inter-particle interactions, while still maintaining a nanopowder form that can be suspended in a biocompatible solvent. These results indicate that the magnetic properties of the synthesized Au-Fe_3_O_4_ nanoparticles are acceptable for biomedical applications.

### 3.2. Transverse Susceptibility Measurements

**Figure 4.**
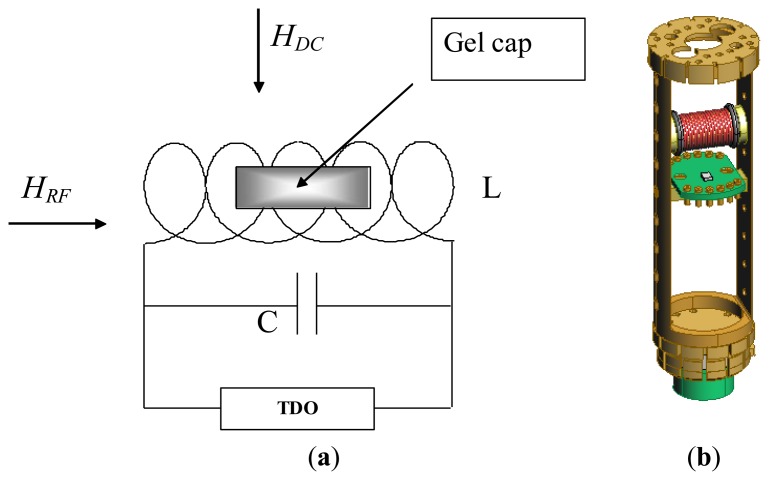
Schematic of the TDO circuit and sample space (**a**) and computer-aided design (CAD) drawing of the inductance coil which serves as the sample holder (**b**).

Since magnetic anisotropy plays a key role in controlling the magnetic properties of magnetic materials, a direct and accurate determination of magnetic anisotropy of the material is a very important measurement [28]. Over the years we have successfully used the radio-frequency (RF) transverse susceptibility (TS) technique, which is based on a very sensitive self-resonant tunnel-diode oscillator (TDO) [29], for directly probing magnetic anisotropy and other fundamental magnetic parameters in systems ranging from thin films and single crystals to nanoparticles [30–33]. In this paper we will show that this technique can be used as a biosensor for detection of biological cells that have taken up magnetic nanoparticles. A simple schematic of the RF transverse susceptibility circuit and a cut-away view of our existing set-up mounted on a cryogenic insert are shown in Figure 4. The TDO is housed outside of a commercial physical properties measurement system (PPMS, Quantum Design) which serves to modulate the applied DC magnetic field (μ_0_H up to ±7 T) as well as the measurement temperature (2 K < T < 300 K). The sample is placed in an inductive coil which is part of an ultrastable, self-resonant tunnel-diode oscillator (operating frequency around 10 MHz to 20 MHz) with a perturbing small amplitude RF field perpendicular to the externally applied DC field (in the PPMS). Transverse susceptibility is defined as $\chi_{t}=\frac{dM_{x}}{dH_{z}}$ and in our experiment this is measured from the shift in resonance frequency as a function of variable field and temperature. Because the change in frequency of the circuit is a direct consequence of the change in inductance as the sample is magnetized, the quantity Δf is directly proportional to Δχ_T_. We are therefore most interested in the quantity:

(1) $$\frac{\Delta \chi_{\text{T}}}{\chi_{\text{T}}}\left(\%\right)=\frac{\left|\chi_{\text{T}}\left(\text{H}\right)-\chi_{\text{T}}^{\text{sat}}\right|}{\chi_{\text{T}}^{\text{sat}}}\times 100$$

as a function of H_DC_ where $\chi_{T}^{\text{sat}}$ is the transverse susceptibility at the saturating field H_sat_. This quantity, which represents a figure of merit, does not depend on geometrical parameters and is useful for comparing the transverse susceptibility data for different samples, or for the same sample under different conditions.

Since this is a technique based on finding the change in the resonant frequency of the circuit in the presence of a magnetic field, the uncertainty in a specific data point is determined by the uncertainty in each resonant frequency measured and the change in each resonant frequency in the presence of a field. It has been theoretically shown that the TS spectrum in a unipolar field scan from positive to negative saturation should consist of three singularities of which two occur at the anisotropy fields (±H_k_) and one at the switching field (±H_s_) [34,35]. However, we have experimentally shown that for an array of nanoparticles with a distribution in size, the switching peak is often merged with one of the anisotropy peaks and a marked asymmetry in both peak location heights can be seen [33]. It is worth mentioning that the anisotropy constant (K) can be extracted from the relation *H_k_* = 2*K*/*M*_S_ where *M*_S_ is the saturation magnetization. Since the high sensitivity comes from our ability to detect changes of the order of a few Hz in the overall resonance of 10 MHz to 20 MHz, the TS technique is well suited for biosensing where the signal from even a small number of target cells that have taken up magnetic nanoparticles can be detected. This technique is also good for evaluating magnetic nanoparticles for MRI contrast enhancement since the set-up geometry is nearly identical.

**Figure 5.**
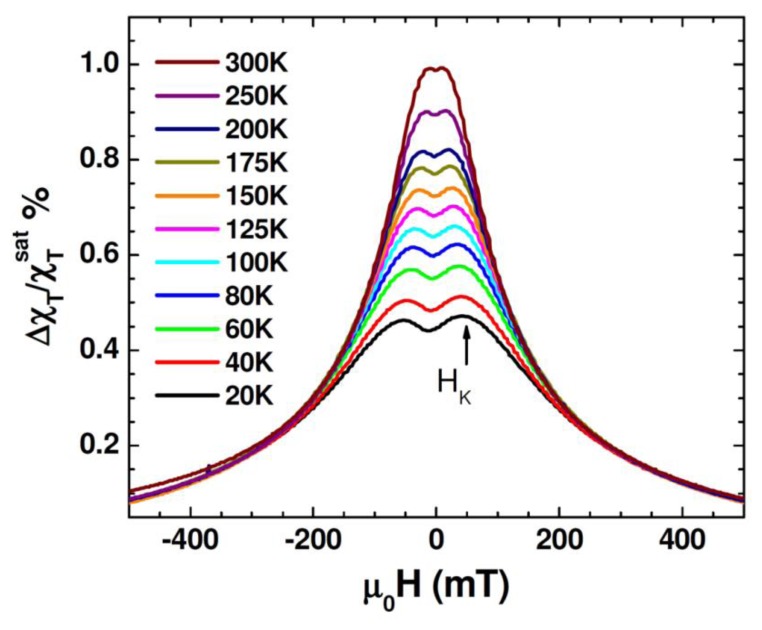
Transverse susceptibility scan taken at different temperatures for Au-Fe_3_O_4_ nanoparticles.

Figure 5 shows unipolar TS scans with magnetic fields sweeping from 0.5 T to –0.5 T for different temperatures between 20 K and 300 K for the Au-Fe_3_O_4_ nanoparticles. It is observed that in the temperature range of 20 K to 300 K the TS profiles show a double-peak and the peak locations (which correspond to ±H_K_) shift to smaller fields as the temperature is increased. This trend can be reconciled with the fact that the particle system transitioned from the ferromagnetic state to the superparametic state [30–33]. However, the presence of H_K_ detected even at 300 K indicates that not all of the Au-Fe_3_O_4_ particles have undergone the ferromagnetic to superparamagnetic transition. This feature will be used to distinguish the difference in TS signal between the cells with and without Au-Fe_3_O_4_ nanoparticles, and the cells containing different amounts of Au-Fe_3_O_4_ nanoparticles. For each sample, TS measurements were performed on cells that were not exposed to nanoparticles, as well as cells after uptake of Au-Fe_3_O_4_ nanoparticles at different concentrations (0.05 mg/mL, 0.1 mg/mL, 0.5 mg/mL, and 1 mg/mL buffer). A sample of cells was placed inside a liquid-safe, 1 mL sample holder.

**Figure 6.**
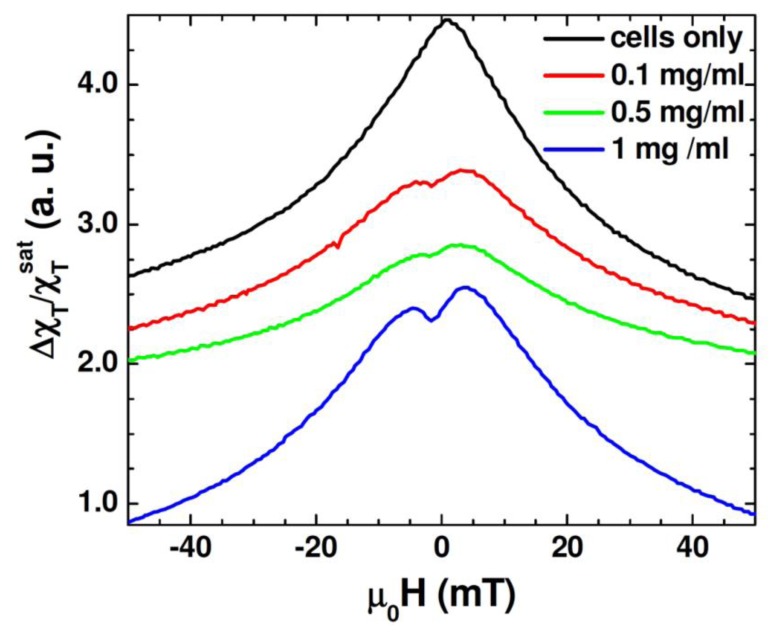
Transverse susceptibility measurements of HEK cells with varying concentrations of Au-Fe_3_O_4_ nanoparticles. The black scan is for the cells without any nanoparticles. The scans have been shifted vertically to better compare the characteristics of each sample.

Figure 6 shows the unipolar TS scan of the cells with several concentrations of the Au-Fe_3_O_4_ nanoparticles, as well as a scan of cells without nanoparticles. It can be seen that the TS probe was able to detect a signal from the nanoparticles inside the cells, whereas the cells by themselves left no signal. While the highest concentration of the Au-Fe_3_O_4_ nanoparticles (1 mg/mL) gives the best signal, it is important to note that at concentrations as low as 0.1 mg/mL, the signal of the Au-Fe_3_O_4_ nanoparticles can still be detected. The anisotropy peaks seen for nanoparticles inside cells appear more defined than those seen at room temperature for Au-Fe_3_O_4_ particles alone. In this case, the same batch of Au-Fe_3_O_4_ particles was used for separate measurement and for uptake. It has been noted that ferromagnetic nanoparticles suspended in a liquid show a different magnetic behavior from that of their dried form [36]. In particular, the dried nanoparticles show a certain value of coercivity, while zero coercivity is found in the suspended nanoparticles due to the additional degrees of rotational freedom of the particles in a liquid. In the present study, a more pronounced asymmetry in TS profiles is seen in the cell samples containing Au-Fe_3_O_4_ nanoparticles (Figure 6) as compared to the Au-Fe_3_O_4_ nanoparticles alone (Figure 5). This feature could be attributed to the inter-particle interactions that were likely weakened in the particle-containing cell samples being diluted in a liquid [32]. However, the appearance of the more defined TS peaks (at ± H _K_) in these samples suggests that the inter-particle interactions alone cannot account for the observed phenomena and that the additional effect of the physical rotation of the particles should also be considered.

Beside these effects, we recall that certain types of cells will only take up particles in a particular size range [5,13], which was why the particles in this experiment were synthesized to be around 60 nm. However, while the DC measurements and even the transverse susceptibility measurements of the particles alone pointed to a size dispersion (D = 60 nm ± 10 nm), it may be that the cells only take up the biggest particles, acting as a size selection mechanism for the nanoparticles. If many of the smaller particles were not taken up by the cells, the ferromagnetic signal at 12 MHz would be stronger in this sample than the polydisperse nanoparticle sample. This experiment demonstrates how transverse susceptibility as a measurement technique can act as a biosensor for the presence of magnetic nanoparticles inside targeted cells. This could be used in a diagnostic capacity if the nanoparticles were functionalized with a biomarker specific to a type of cancer cell. Targeting and uptake of the nanoparticles would only occur if the cells were cancerous. Transverse susceptibility could then be used to determine if the cells had taken up the particles and hence were cancerous. While the PPMS was used to provide the H_DC_, it is well worth noting that the fields needed for this experiment are less than 50 mT, a field strength easily achievable with an electromagnet. The measurements were also taken at room temperature and not in a vacuum. Realistically, transverse susceptibility used in this capacity could be set up as a table-top experiment rather than integrated into the commercial PPMS.

## 4. Conclusions

We have shown that the radio-frequency transverse susceptibility technique can be used in a biosensor for detection of human embryonic kidney cells that have taken up Au-Fe_3_O_4_ nanoparticles. This technique is highly sensitive for two distinct reasons: any susceptibility measurement is a measure of the derivative of the magnetic response with respect to field, and our method of measurement is a resonant technique that can detect changes in frequency on the order of 10 Hz in 10 MHz. It was found that even a small number of cells that have taken up magnetic nanoparticles when placed inside the sample space of the transverse susceptibility probe can yield a signal characteristic of the magnetic nanoparticles. This technique can be used in a diagnostic capacity if the nanoparticles are functionalized with a biomarker specific to a type of cancer cell. This technique can also be good for evaluating magnetic nanoparticles for MRI contrast enhancement since the set-up geometry is nearly identical.
